# Advancing Timberline on Mt. Fuji between 1978 and 2018

**DOI:** 10.3390/plants9111537

**Published:** 2020-11-10

**Authors:** Hitoshi Sakio, Takehiro Masuzawa

**Affiliations:** 1 Sado Island Center for Ecological Sustainability, Niigata University, Sado 952-2206, Japan; 2 Department of Biology, Faculty of Science, Shizuoka University, Shizuoka 422-8529, Japan; masuzawa.takehiro@shizuoka.ac.jp

**Keywords:** alpine timberline, global warming, *Larix kaempferi*, long-term ecological research, Mt. Fuji, seedling

## Abstract

Climate change is a major cause of changes in alpine and polar vegetation, particularly at the edges of distributions. In temperate regions, these changes are expected to occur at the timberline of alpine zones. On Mt. Fuji, the highest mountain in Japan, the timberline is located 2400–2500 m above sea level. Over a 40-year period (1978–2018), we researched changes in the timberline vegetation of Mt. Fuji. A permanent belt transect extending from the upper timberline to subalpine zones was set up in August 1978. Tree diameters and heights were recorded at the establishment of the transect and every 20 years afterwards. Over the 40 years of the study, the timberline advanced rapidly upwards, and the degree of vegetation cover above the timberline increased remarkably. Notably, the expansion of *Salix reinii* into the upper part of the timberline facilitated the subsequent spread of *Larix kaempferi* into this zone. Seedlings of *L. kaempferi* were particularly abundant at the upper timberline and became established on the uppermost part of the slope. The shape of *L. kaempferi* at the upper timberline changed from a prostrate form to an upright tree form. We conclude that the upward advance of the alpine timberline observed on Mt. Fuji is due to climate change.

## 1. Introduction

Global climate change has been analyzed using long-term meteorological and oceanographic data. Over the period from 1880 to 2012, globally averaged combined land and ocean surface temperatures followed a linear trend, increasing by 0.85 °C [1]. At the end of the 21st century (2081–2100), the change in the global surface temperature relative to 1850–1900 is projected with high confidence to exceed 1.5 °C under RCP (Representative Concentration Pathway) 4.5, RCP6.0, and RCP8.5 scenarios [1]. Breeding and species selection in agriculture have had to adapt to global warming, and the spread of infectious plant diseases associated with global warming has become a major problem [2]. In addition, glacial retreat has had considerable effects on ecosystems [3] and on the distribution of organisms in both polar regions [4] and alpine zones [5]. The movement of plant communities to the north has been confirmed in polar regions in the Northern Hemisphere, and the early arrival of spring has caused a mismatch in plant pollination, even in temperate regions [6]. Plant communities have also been spreading upwards in alpine zones [7]. Long-term monitoring is needed to confirm whether these changes are temporary or permanent, and to verify whether the simulations based on past data are correct. Long-term monitoring research is ongoing worldwide [8]. In Japan, long-term monitoring observations have been conducted in forests, grasslands, lakes, marshes, and oceans nationwide since 2003 through the “Monitoring Site 1000” project of the Ministry of the Environment [9]. Because a 20-year period is too short to capture the effects of global warming on long-term changes in natural ecosystems, these projects are expected to continue in the future.

The alpine timberline is a forefront of struggle for tree survival [10]. In this zone, the timberline migrates upwards or downwards in response to plant-limiting factors, such as low air temperature, frost damage, carbon limitation, winter desiccation, and strong wind [11]. In particular, the area called the “kampfzone” is the place with the most dynamic changes in the timberline ecosystem [10]. This kampfzone is characterized by extreme ecological conditions for survival, growth, and competition [12], and is very sensitive to changing climatic conditions. Long-term observation of ecosystem changes in such places is therefore considered useful for clearly understanding the effects of global climate change on ecosystems.

Mt. Fuji is the highest mountain with a timberline in Japan. Timberline zones of most high mountains in Japan are dominated by *Pinus pumila* communities [13,14]. In Europe and North America, coniferous trees in the upper part of the timberline exhibit a high degree of phenotypic plasticity in reaction to environmental factors in the kampfzone [10], and the forest structure around the timberline in these regions is clearly different from that of Japan. Unlike other high mountains in Japan, Mt. Fuji lacks *P. pumila* and has a forest structure similar to the timberlines of Europe and North America. Conducting a survey on Mt. Fuji is important to allow comparisons of timberline dynamics in mid-latitude Japan with those in Europe [15,16,17,18,19,20] and North America, and to analyze their relationship with global warming. In many locations in Europe, humans have had a long-term impact on the timberline, especially in the 17th to 19th centuries when high mountain meadows were extensively used for grazing and haymaking [21,22,23]. However, the timberline on Mt. Fuji has always been maintained in a natural state, with the exception of some low-impact activities such as mountain climbing. For these reasons, investigating the timberline of Mt. Fuji is especially important compared with other high mountains in Japan.

Many research reports have appeared on forest vegetation on Mt. Fuji, and the upward movement of the timberline has been pointed out in previous studies [24,25,26]. Oka [26] confirmed this phenomenon based on field surveys and an annual ring analysis, and Maruta and Masuyama [25] reported similar observations from a time series analysis using aerial photographs. These studies were short term, however, and did not clarify the mechanism and dynamics of forest change. No long-term detailed studies of the timberline of Mt. Fuji had thus been conducted. In 1978, we installed a permanent quadrat at the timberline of Mt. Fuji and have been continuously tracking the dynamics and mechanisms of the forest vegetation [27,28]. In the early years of our study, we found that the timberline had expanded upwards considerably between 1978 and 1999 [28], possibly because of climate change.

The purpose of this study was to clarify how vegetation at the timberline of Mt. Fuji changed during the 40 years from 1978 to 2018. In particular, we aimed to determine (1) whether the observed upward movement of the timberline of Mt. Fuji is continuing and (2) whether the forest structure of the timberline has changed over this period. Our results may be useful for predicting how the recent temperature rise will affect vegetation in the timberline of Mt. Fuji and how global warming will impact forest vegetation in extreme environments.

## 2. Results

### 2.1. Change in Timberline Vegetation over Time

**Figure 1:**
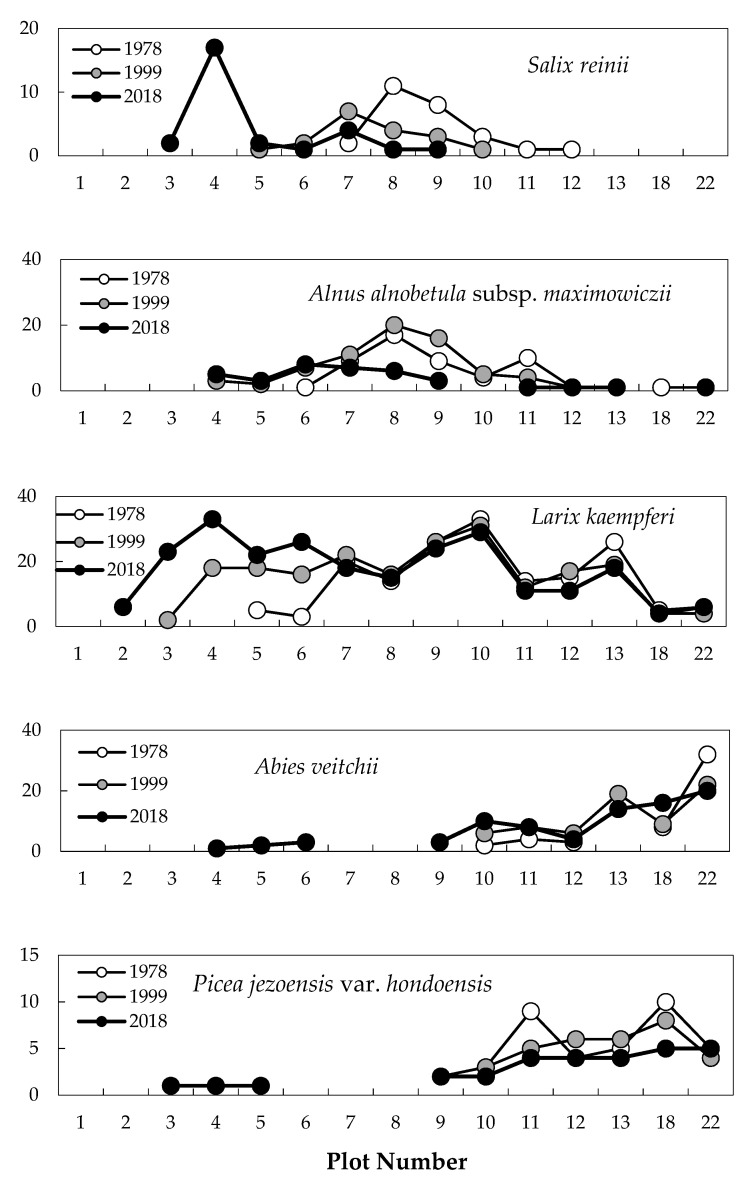
Distribution of the number of trees across the slope gradient between 1978 and 2018. White, gray, and black circles are the number of trees in 1978, 1999, and 2018, respectively.

The timberline vegetation of Mt. Fuji fluctuated drastically during 1978–2018. The number of trunks with a height of 130 cm or more varied greatly in *Alnus alnobetula* subsp. *maximowiczii*, *Salix reinii*, and *Larix kaempferi* (Figure 1). *S. reinii* decreased sharply in plots 8 and 9, and all trunks disappeared in plots 10–12 in 2018. Conversely, this species was not seen at all in plots 3 and 4 in 1978, but increased in these plots to 2 and 17 trunks, respectively, in 2018. *A. alnobetula* subsp. *maximowiczii* showed little change in plots 8–11 between 1978 and 1999 but had declined sharply by 2018. This species also increased slightly in plots 4–6 between 1978 and 2018 (Friedman’s test, *p* < 0.05). *L. kaempferi* gradually increased in plots 5 and 6 from 1978 to 2018. This species first appeared in plots 3 and 4 in 1988 and in plot 2 in 2018. The number of *L. kaempferi* individuals rapidly increased in plots 3 and 4 between 1999 and 2018, and significantly increased in plots 2–6 between 1978 and 2018 (Friedman’s test, *p* < 0.01). This species remained constant in plots 7–22. Few changes were observed in *Abies veitchii* and *Picea jezoensis* var. *hondoensis* in any plots during the research period.

**Figure 2:**
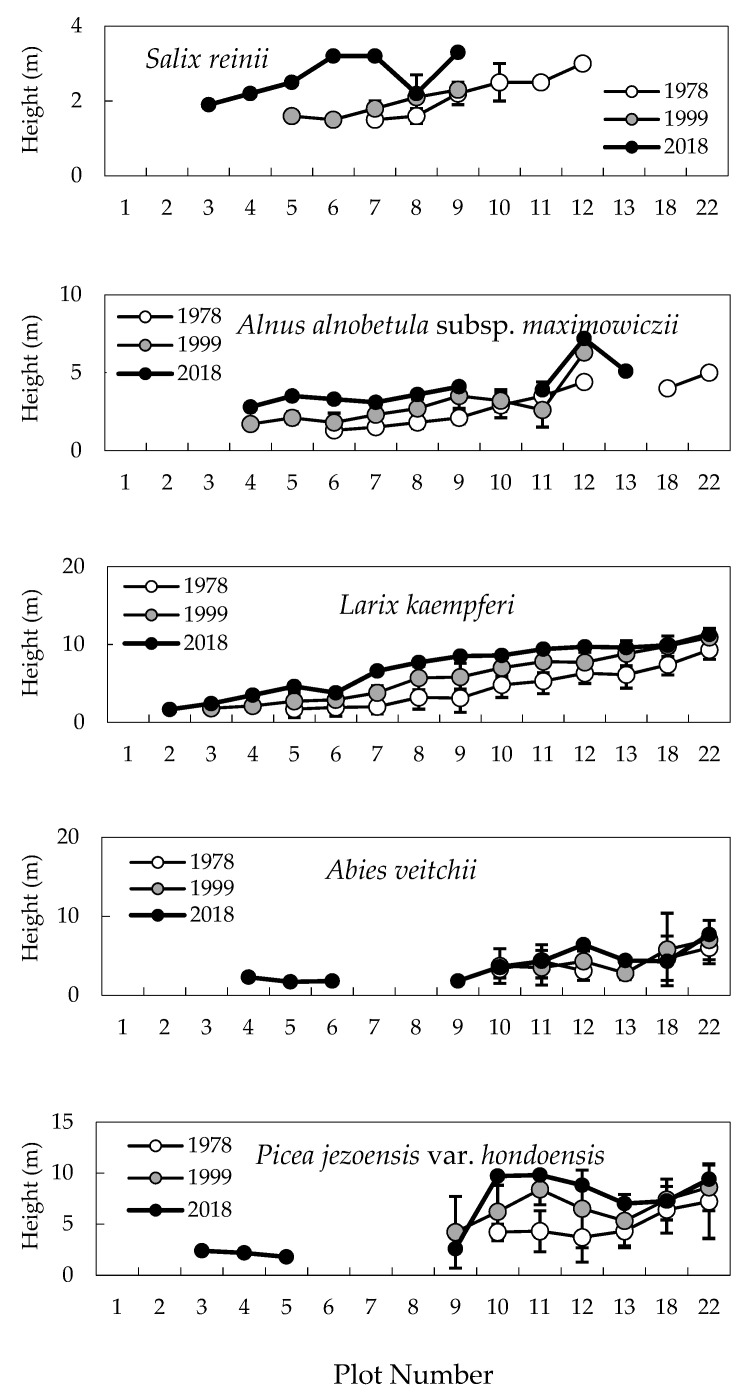
Distribution of tree heights across the slope gradient between 1978 and 2018. White, gray, and black circles are tree heights in 1978, 1999, and 2018, respectively.

The average heights of *S. reinii* and *A. alnobetula* subsp. *maximowiczii* increased in plots 7–9 from 1978 to 2018 (Figure 2). The average heights of *S. reinii* and *A. alnobetula* subsp*. maximowiczii* significantly increased between 1978 and 2018 in plots 3–6 and 4–6, respectively (Friedman’s test, *p* < 0.05). In the case of *L. kaempferi*, average tree heights increased continuously in all study plots, with significant increases observed in plots 2–6 from 1978 to 2018 (Friedman’s test, *p* < 0.01). In 2018, new trunks of *A. veitchii* and *P. jezoensis* var. *hondoensis* appeared in plots 4–6 and 3–5, respectively.

**Figure 3:**
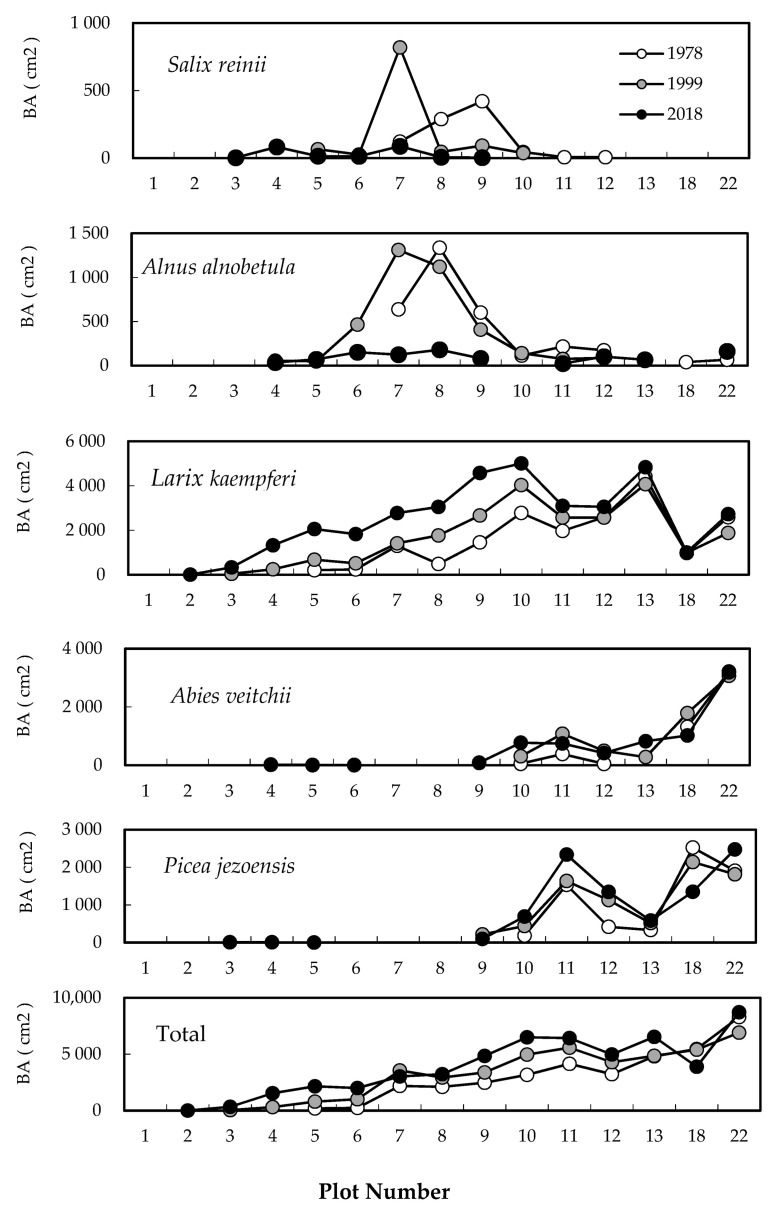
Distribution of tree basal areas (BAs) across the slope gradient between 1978 and 2018. White, gray, and black circles are BAs in 1978, 1999, and 2018, respectively.

The total basal areas (BAs) for all species significantly increased over the 40-year study period, except in plots 7 and 18 (Figure 3; Friedman’s test, *p* < 0.001). The BA of *A. alnobetula* subsp. *maximowiczii* and *S. reinii* increased at elevations above plot 7 (at the timberline) and decreased below plot 8 from 1978 to 1999. *A. alnobetula* subsp. *maximowiczii* and *S. reinii* exhibited 6.7- and 2.1-fold increases, respectively, in plot 7 between 1978 and 1999, but the BA of these two species decreased sharply in all plots in 2018. The BA of *L. kaempferi* significantly increased in all plots from 1978 to 2018 (Friedman’s test, *p* < 0.0001). The BA of *L. kaempferi* increased at a similar rate between 1978–1999 vs. 1999–2018 in plots 8, 9, 10, and 11 but increased more rapidly in plots 4–7 between 1999 and 2018.

### 2.2. Difference in Changes between the Two 20-Year Periods

**Figure 4:**
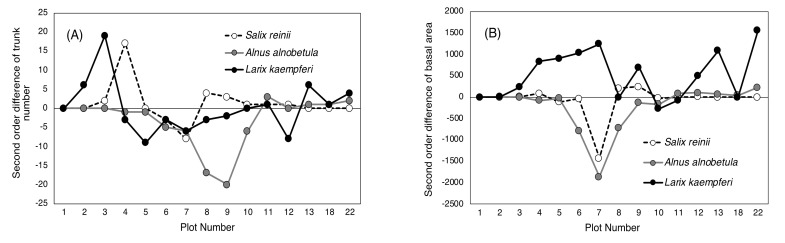
Second-order differences among three measurements (1978, 1999, and 2018) of trunk number (**A**) and basal area (**B**) of major tree species.

Changes between the two 20-year periods are illustrated by the second-order difference between 1978, 1999, and 2018 (Figure 4). The number of trunks of *S. reinii* and *L. kaempferi* increased in plots 4 and 3, respectively, between the two periods, while *A. alnobetula* subsp. *maximowiczii* decreased in plots 8 and 9. The BA of *S. reinii* and *A. alnobetula* subsp. *maximowiczii* decreased in plot 7 between the two periods*,* whereas that of *L. kaempferi* increased markedly in plots 4–7.

### 2.3. Establishment of Seedlings at the Upper Timberline

**Figure 5:**
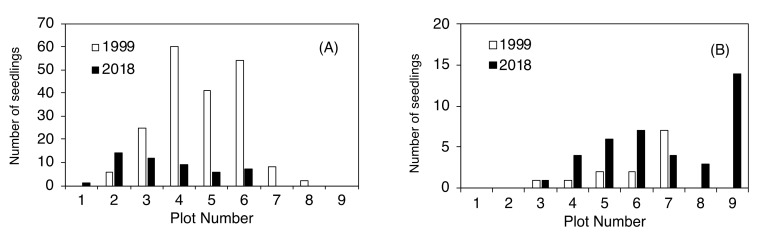
Number of established seedlings of *Larix kaempferi* (**A**) and *Abies veitchii* (**B**) as of 1999 and 2018.

*L. kaempferi* seedlings were widely distributed throughout the upper area of the timberline. In particular, seedlings had colonized plots 3–6 in 1999 but decreased over the next 20 years (Figure 5A). Between 1999 and 2018, *L. kaempferi* disappeared from plots 7 and 8, and the total number of seedlings decreased by 19% in plots 3–6. In contrast, the number of *L. kaempferi* seedlings in plot 2 increased from 6 to 14 between 1999 and 2018, and seedlings were established in plot 1 for the first time during this period. Seedlings of *A. veitchii* invaded vegetation patches in plots 3–7 in 1999; this species had spread to plots 8 and 9 by 2018, and the number of seedlings in plots 3–6 had increased (Figure 5B).

Many seedlings were established in the upper timberline area. In the case of *L. kaempferi,* 196 and 12 new seedlings were established from 1978–1999 and 1999–2018, respectively (Table 1). The number of new seedlings during the first 20-year period was significantly higher than during the latter (Wilcoxon signed-rank test, *p* < 0.05). The range of newly invaded plots was moving upwards. No seedlings were established in plots 6–8 between 1999 and 2018. Seedlings recorded in plot 2 between 1999 and 2018 were clearly taller than those established between 1978 and 1999. In the case of *A. veitchii,* 17 and 12 new seedlings were established from 1978–1999 and 1999–2018, respectively (Table 2). The range of established new seedlings was the same in both periods.

**Figure 6:**
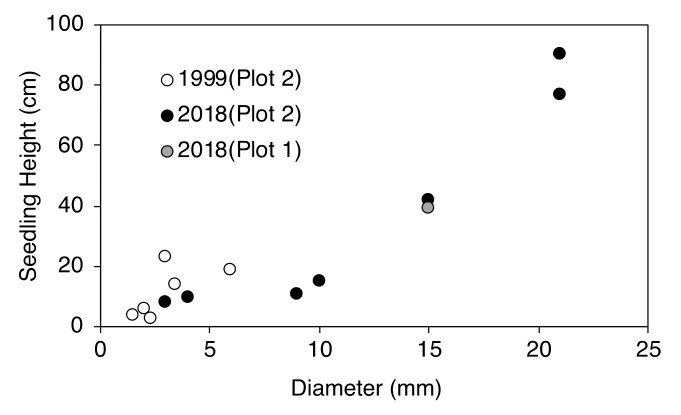
Relationship between the diameter and height of seedlings in the upper kampfzone. White circles are seedlings established as of 1999 in plot 2. Black and gray circles are seedlings established as of 2018 in plots 2 and 1, respectively.

In the period 1978–1999 and 1999–2018, *L. kaempferi* individuals newly established above the timberline varied greatly in size (Figure 6). The heights of all newly established seedlings were less than 20 cm between 1978–1999 but up to 90 cm between 1999–2018. Similar to seedling heights, the diameter at ground surface of seedlings established between 1999 and 2018 was larger than that of those established between 1978 and 1999 (Welch’s *t*-test, *p* < 0.01).

### 2.4. Degree of Vegetation Cover

**Figure 7:**
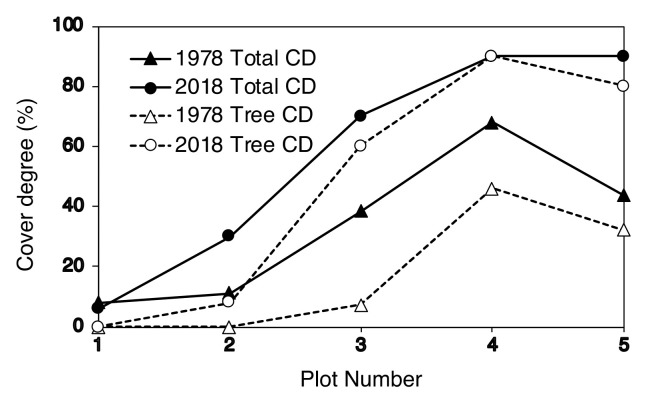
Degree of vegetation cover in 1978 and 2018. The total degree of cover (CD) includes the degree of tree cover.

Changes in the degree of vegetation cover of the upper timberline area are shown in Figure 7. The total degree of vegetation cover increased from the top to the bottom of the upper timberline in 1978. During the 40 years, this value did not change in plot 1 but increased greatly in plots 2–5. The degree of tree cover also increased during the 40 years. No change was observed in plot 1, but the degree of tree cover increased in plots 2–5, especially in plots 3–5. In 2018, trees accounted for approximately 90% of the total vegetation cover in plots 3–5. As a result, most of the vegetation in plots 3–5 was dominated by trees in 2018.

## 3. Discussion

According to our earlier findings, the timberline of Mt. Fuji moved upwards between 1978, when the research site was set up, and 1999 [27,28]. In the present study, we found that the timberline of Mt. Fuji continued to considerably advance upwards until by 2018.

The vegetation around the Mt. Fuji timberline varies according to elevation, with the change in vegetation type from upper to lower elevations following this order: herbaceous plant patches, deciduous shrubs (*S. reinii*, *L. kaempferi*, and *A. alnobetula subsp. maximowiczii*), deciduous *L. kaempferi* forests, and evergreen coniferous forests of *A. veitchii* and *P. jezoensis* var. *hondoensis* [27]. The most striking change over the 40 studied years was that of the upper timberline vegetation (plots 2–6) above the deciduous shrubs (Figure 2 and Figure 3). In plot 1, at the top, no change was observed in vegetation cover over 40 years, whereas vegetation cover in plots 2–5 increased considerably, especially that due to woody plants (Figure 7). This change was the result of an increase in the number of *S. reinii* and *L. kaempferi* individuals. Both species have pioneering properties and can invade bare land, but they have different life forms. *S. reinii* is a bush with multiple stems and a maximum height of 3 m. In contrast, *L. kaempferi* can form forests more than 10 m high below the timberline (Figure 4) and shade out *S. reinii* individuals during growth. Although *S. reinii* was able to invade the upper timberline and increase in height (Figure 4), this species was suppressed below the timberline by *L. kaempferi*, and the population therefore decreased sharply (Figure 3 and Figure 5). Conversely, *L. kaempferi* did not decrease in population size after invading and establishing itself at higher elevations, and its BA increased with increasing tree height (Figure 4 and Figure 5). In our earlier study, *L. kaempferi* seedlings were found to be established very close to the edges of vegetation patches [28]. Patches of *S. reinii* may play an important role in the establishment of *L. kaempferi* at the krummholz limit on Mt. Fuji [29,30] and shrubs provide safe sites through creating a more favorable microclimate [31,32]. *S. reinii* may also contribute to tree succession by providing adjacent late colonizers (*L. kaempferi*) with compatible ectomycorrhizal (ECM) symbionts [33].

In contrast to *S. reinii*, *A. alnobetula* subsp*. maximowiczii* markedly decreased, both in terms of population size and BA, over the 40 years without invading upper elevations (Figure 3 and Figure 5). Previous studies have shown that individuals of *A. alnobetula* subsp*. maximowiczii* on Mt. Fuji have a high production rate because of their high photosynthetic rate [34] and the high nitrogen content of leaf litter [35]. In one study, in addition, the amount of annual nitrogen fixation by nodules was found to be almost the same as that of nitrogen used for annual growth [36]. This species has therefore been considered to contribute to the upward movement in nitrogen supply at the timberline of Mt. Fuji [28]. The rapid decline of *A. alnobetula* subsp. *maximowiczii* dwarf forests over the past 40 years, however, suggests that factors other than improvements in soil nitrogen have had a major effect on the advance of the timberline on Mt. Fuji.

In the case of evergreen conifers, seedlings of *A. veitchii* and *P. jezoensis* var. *hondoensis* were present in the upper part of the timberline in 1999. By 2018, some individuals with a height of approximately 2 m were observed at this higher elevation (plots 3–6). These individuals had developed from the seedlings established in 1999 (Figure 3).

Seedling establishment is an important factor in the expansion of plant and forest distributions. Many seedlings of species such as *L. kaempferi*, *A. veitchii*, and *P. jezoensis* var. *hondoensis* were found at the upper timberline (plots 3–6) between 1978 and 1999 [28]. In particular, *L. kaempferi* invasion and establishment was extensive, with a total of 196 seedlings found in 1999 in plots 2–8 (Table 1). By 2018, however, many seedlings had died as a result of an increase in the vegetation cover at the upper timberline (plots 3–6). Although 12 new seedlings were established between 1999 and 2018 (Table 1), the overall population decreased to 49 in 2018 (Figure 7). The seedling population continued to increase in the uppermost areas (plots 1 and 2), however, as *L. kaempferi* was first established in plot 1 and doubled in plot 2 in 1999 (Figure 7). The sizes of *L. kaempferi* individuals established between 1978–1999 and 1999–2018 were clearly larger in the latter period in plot 2 (Table 1). As described above, seedlings of *L. kaempferi* were steadily advancing above the timberline, which is considered to be a more severe environment.

Seedlings of *A. veitchii*, which has a higher shade tolerance than *L. kaempferi*, were found to be distributed throughout the timberline ecotone (plots 3–9), and the number of *A. veitchii* seedlings had increased in 2018 compared with 1999 (Figure 7). The number of individuals established between 1999 and 2018 was almost the same as between 1978 and 1999 (Table 2). In other words, *A. veitchii* is a recent invader of locations of previous *L. kaempferi* invasion and growth. *L. kaempferi* thus acts as a facilitator for *A. veitchii*, as the former has deeper roots than the latter and can avoid desiccation [37,38].

**Figure 8:**
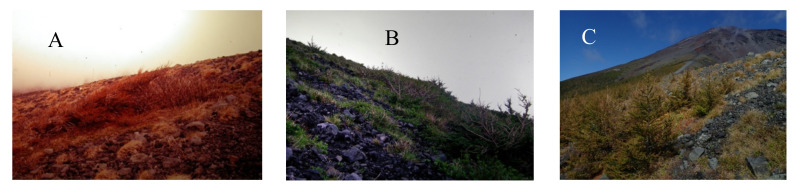
Tree shape of *L. kaempferi* at the timberline of Mt. Fuji. (**A**) Prostrate trees at the upper timberline in plots 2 and 3 in 1978. (**B**) Prostrate trees with erect stems in plot 5 in 1978. (**C**) Seedlings with erect stems in plots 2 and 3 in 2018.

Tree forms are shaped by physical forces, such as strong wind and heavy snow, under severe environments [10,21,39,40,41]. The area around the timberline is strongly affected by strong winds in winter. Life forms with highly variable physiognomy predominate among woody plants, ranging from bushes with “flagged” leaders to cushions of krummholz pressed close to the ground (table shape) [10]. Prostrate *L. kaempferi* at the upper timberline in 1978 (plots 3 and 4) are shown in Figure 8A, while Figure 8B shows prostrate *L. kaempferi* with erect stems and an estimated age of 150 years (plot 5) at that time. In 2018, however, a different landscape of tree shapes was evident (Figure 8C). In particular, *L. kaempferi* that had newly invaded the upper portion of the krummholz limit were growing with erect trunks without dwarfing (plots 2 and 3; Figure 8C). Maruta and Masuyama [25] have reported that the first step in advancing timberline is the establishment of the dwarf type of *L. kaempferi*, which contrasts with individuals at lower elevations that gradually form erect trunks. The conflict between the results of their study and our findings may be due to differences in topography between the respective research sites as well as factors related to climate change, such as an increase in temperature.

**Figure 9:**
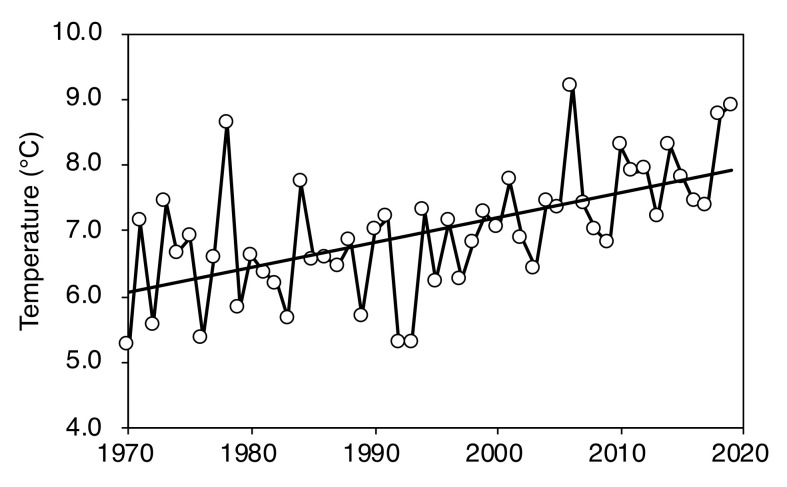
The average maximum temperature between June and September at the summit of Mt. Fuji over 50 years. The data in this figure are from the Japan Meteorological Agency (http://www.jma.go.jp/jma/menu/report.html).

Air temperature and CO_2_ concentration are important determinants of plant growth. Global warming has recently become a problem [1]. The significant warming occurring in recent years may have changed timberline ecosystems in Europe [7,42,43,44], China [45], and Japan [28]. During the last 40 years, the average maximum temperature has continued to rise during the plant growth period on Mt. Fuji (Figure 9). Higher temperatures will extend the plant growth period and elevate photosynthetic rates. As the photosynthetic period lengthens and the photosynthetic rate increases, the annual growth rate may increase, and shoots may be formed that can better withstand the winter environment. In addition to air temperature, the CO_2_ concentration is rising. The mean CO_2_ concentration at the summit of Mt. Fuji was approximately 335 ppm in August 1981 [46], 388 ppm in 2010 [47], and above 400 ppm in 2015 [48]. The saturation limit for CO_2_ assimilation in *L. kaempferi* is at an intercellular CO_2_ concentration of 600 ppm, regardless of mineral nutrient supply [49]. The photosynthetic rate may therefore continue to rise. As mentioned above, the temperature rise and the increase in CO_2_ concentration are considered to be factors that increase the biomass production of trees at the timberline. As a result, the annual growth of *L. kaempferi* may have increased, and erect shoots may be able to survive, even in the severe winter environment, without unusual phenotypic response.

The mechanism responsible for timberline rise on Mt. Fuji has been thought to entail the invasion of deciduous shrub trees, such as *S. reinii* and *A. alnobetula* subsp. *maximowiczii*, into herbaceous patches to form shrub forests, with *L. kaempferi* also invading to form a table-shaped shrub forest that eventually stands upright. However, *L. kaempferi* has invaded the upper part of the timberline and continued to grow upright without forming dwarf shrubs with a prostrate form. This phenomenon is thought to be due to changes in external factors in addition to natural succession occurring after the eruption in 1707. One such external factor is an increase in annual growth due to temperature rise. This temperature increase promotes an increase in photosynthetic rate and an expansion in the photosynthetic period. In addition to the rise in air temperature, the increase in CO_2_ concentration accelerates the growth rate.

Previous studies have pointed out the consequences of the imbalance between rapid climate change and slow biological responses. Even among the most mobile species such as butterflies, these pollinators have been unable to extend their ranges as fast as required to keep pace with climate change [50,51]. On the other hand, results of our research over forty years and global warming forecasts [1], has suggested that the timberline of Mt. Fuji will continue to advance upwards. These results may indicate that monitoring of the alpine ecosystems may be effective in capturing the sensitive impact of climate change on forests. The “Monitoring site 1000” project of the Ministry of the Environment in Japan, which began in 2003, has yielded results for many forests, but its impact on rapid climate change in recent years has been less apparent. Therefore, long-term monitoring in various climate zones, including alpine ecosystems, will be necessary to assess the effects of global warming on organisms.

## 4. Study Site and Methods

### 4.1. Study Site

Mt. Fuji (3776 m) is the highest volcano in Japan. A stratovolcano mainly composed of basalt, the mountain spans both Yamanashi and Shizuoka prefectures. Mt. Fuji is still a young adolescent volcano and is believed to have begun on a submarine volcano one million to 700,000 years ago, but its exact origins remain unclear. The hillside over 2500 m above sea level is covered with volcanic products, with vegetation distributed on the slope below. The research site (35°21′ N, 138°45′ E) was located at the timberline (ca. 2400 m) on the southeastern slope, where the vegetation is recovering from damage caused by the 1707 volcanic eruption of Hoei-Zan, a parasitic crater. This area is a special protection zone of Fuji-Hakone-Izu National Park. Special protection areas, which feature the most outstanding natural scenery and pristine conditions, are the most strictly regulated areas of the park.

**Figure 10:**
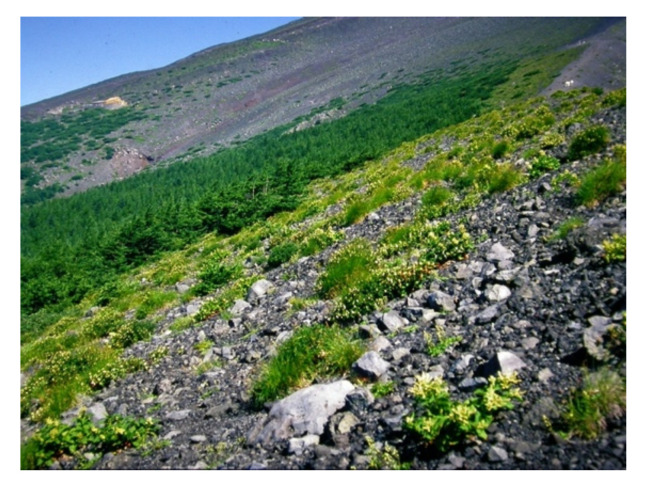
Timberline vegetation on Mt. Fuji in 1980.

Most of the timberline vegetation in Japan comprises of *Pinus pumila*, whereas the timberline on Mt. Fuji is dominated by *L. kaempferi.* The vegetation at the timberline of Mt. Fuji changes dramatically as one proceeds down the slope, with variations in herbs, deciduous shrubs, deciduous conifers, and evergreen conifers (Figure 10). Perennial herb vegetation occurs above the timberline and includes *Astragalus laxmannii* var. *adsurgens*, *Arabis serrata* var. *serrata*, *Aconogonon weyrichii* var. *alpinum*, *Carex doenitzii*, and *Artemisia pedunculosa* [27]. The timberline vegetation comprises of deciduous dwarf trees: *A. alnobetula* subsp*. maximowiczii*, *S. reinii*, *L. kaempferi*, and *Betula ermanii*. Downslope of the dwarf vegetation, the forest composition changes from *L. kaempferi* forest to coniferous evergreen forest, of which *A. veitchii* and *P. jezoensis* var. *hondoensis* are dominant [27].

The timberline weather on Mt. Fuji is very cold and windy [52], but little snow cover is present (ca. 30 cm in depth from November to February). The annual mean air temperature is 1.1 °C, with the highest and lowest monthly means of 11.8 °C in August and −9.5 °C in February [27]. The annual precipitation is approximately 4500 mm [53]. The precipitation level is high throughout the year, especially during the summer growing season because of the rainy and typhoon season. Relative humidity is very high because of frequent fog from June to September (mean >80%) [27].

The surface substratum at the research site consists of basalt scoria from the volcanic eruptions of Hoei in 1707. This scoria is easily moved downward by repeated freezing and thawing, and by strong wind or heavy rain. The ground surface is therefore very unstable. The nitrogen and carbon content of the soil at the upper timberline is very low, 0.02% and 0.3%, respectively [27].

### 4.2. Methods

**Figure 11:**
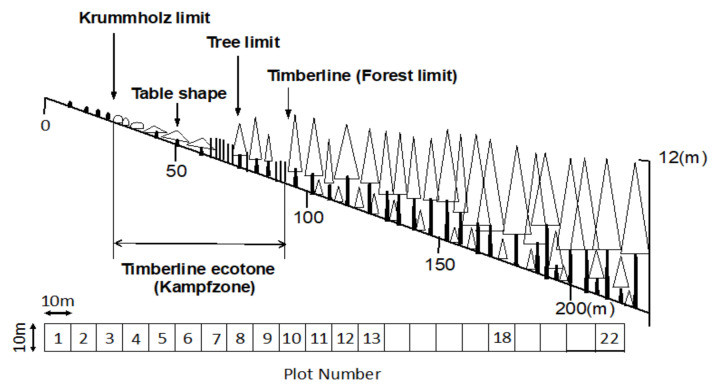
Forest profile of the timberline on Mt. Fuji. Dominant species in many perennial herb patches at the upper krummholz limit are *Aconogonon weyrichii* var. *alpinum*, *Carex doenitzii*, *Hedysarum vicioides* subsp. *japonicum*, and *Arabis serrata* var. *serrata*. White triangles in the canopy indicate *Larix kaempferi*, and gray triangles represent evergreen coniferous trees (*Abies veitchii* and *Picea jezoensis* var. *hondoensis*). The diagram at the bottom of the figure depicts the layout of the research plots.

We established a 220-m-long permanent belt transect (10 m wide) extending from the upper timberline zone to the subalpine forest dominated by coniferous evergreen trees in August 1978. The transect comprised of 22 numbered contiguous plots (10 × 10 m; Figure 11). All living trees (≥130 cm tall) were identified to species level. The diameter at breast height (DBH; diameter 130 cm above ground level) and height of trees were recorded in 1978 in the uppermost plots at 0–130 m along the transect and in lower plots at 180 and 220 m [27]. For deciduous shrub tree such as *A. alnobetula* subsp. *maximowiczii* and *S. reinii*, the longest trunks of individual plants were selected for tree height measurements. Basal area (BA) was calculated from the DBH data for all trunks and summarized as the total area per plot for each tree species. A second census taken in 1999 confirmed that the timberline of Mt. Fuji was advancing upwards [28]. At that time, climate change was proposed to be one of the causes of this expansion. Since the beginning of the 21st century, extreme weather events have occurred around the world, and rising temperatures have been observed in Japan. To be able to compare changes in the timberline over the next 20 years, we repeated the measurements in 2018. Friedman’s test (followed by a least significant difference test) was used to detect significant differences in the number of trees, tree height, and BA among the three measurements obtained from the very uppermost plots (plots 1–6). In addition, we compared changes in the number of trees and BA between the first half of the study period (1987–1999) and the second half (1999–2018). We first determined the first-order difference between 1987 and 1999 and that between 1999 and 2018. The second-order difference, obtained by subtracting the former from the latter, was then calculated, and the change between the two periods was compared.

The number and height of living *L. kaempferi* seedlings in the nine uppermost plots (1–9) were measured in 1999 and 2018. The number, diameter, and height of new seedlings established between 1978–1999 and 2000–2018 were measured in the nine uppermost plots (1–9) in 1999 and 2018. These data were used to investigate the upwards advance of vegetation.

The total degree of vegetation cover and degree of tree cover in each plot were measured in 1978 and 2018. The degree of vegetation cover, an index of the degree of foliage overgrowth, is defined as the vegetation area obtained by orthographically projecting the foliage of the vegetation onto a horizontal plane, that is, the occupancy degree of vegetation per unit area of each plot. The degree of tree cover is defined in the same way.

## Figures and Tables

**Table 1:** No. and height of *Larix* seedlings established.

Plot Number	No. of New Seedlings		Seedling Height (cm)	
	1978–1999	1999–2018	1978–1999	1999–2018
1	0	1	-	39
2	6	7	12 ± 8	36 ± 14
3	25	2	32 ± 38	18
4	60	1	52 ± 60	80
5	41	1	74 ± 79	11
6	54	0	77 ± 74	-
7	8	0	44 ± 23	-
8	2	0	11 ± 9	-
9	0	0	-	-

**Table 2:** No. and height of *Abies* seedlings established.

Plot Number	No. of New Seedlings		Seedling Height (cm)	
	1978–1999	1999–2018	1978–1999	1999–2018
1	0	0	-	-
2	0	0	-	-
3	1	1	16	96
4	1	2	115	75 ± 45.3
5	2	4	29 ± 16	51.3 ± 36.7
6	2	1	23 ± 9.9	50
7	7	3	57.7 ± 76.3	43.3 ± 20.7
8	4	1	40 ± 23.6	78
9	0	0	-	-
